# Phosphinate-containing heterocycles: A mini-review

**DOI:** 10.3762/bjoc.10.67

**Published:** 2014-03-27

**Authors:** Olivier Berger, Jean-Luc Montchamp

**Affiliations:** 1 Department of Chemistry, Box 298860, Texas Christian University, Fort Worth, Texas 76129, USA

**Keywords:** amino acid, heterocyle, organophosphorus, phosphinic, phosphorus

## Abstract

This review provides an overview of recent efforts towards the synthesis of phosphinate heterocycles R^1^R^2^P(O)(OR). Our laboratory and others’ have been involved in this field and as a result new P–C, P–N, and P–O containing heterocyclic motifs are now available through a variety of methods. While developing rapidly, this area is still in its infancy so that biological testing of the compounds has not yet been conducted and applications are rare. The growing availability of synthetic methods will undoubtedly change this situation in the near future.

## Introduction

The preparation of P-heterocycles has been the subject of many studies over the years, and the field has been extensively reviewed [1–8]. Typically, accessing *P*-heterocycles involves multistep sequences with low overall yields [1–8]. In the past 20 years, significant effort has been devoted to synthetic and reactivity studies of a particular family of organophosphorus compounds: the phosphinates R^1^R^2^P(O)(OR) [9]. Because the phosphinic acid moiety P(O)OH can mimic carboxylic acids, its incorporation into heterocycles may offer new opportunities for the discovery of biologically active analogs. However, little or no biological data is available at this time. Selected recent synthetic work by us and others is presented below.

## Review

### Phospholes

**Scheme 1:**
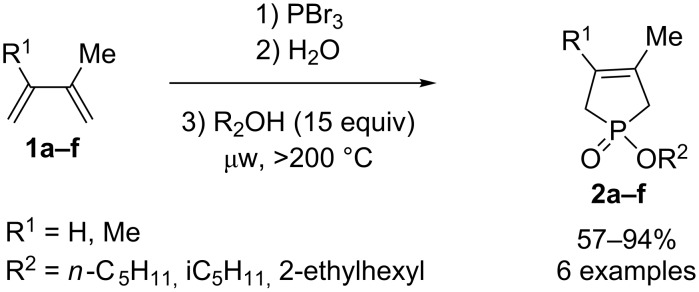
McCormack synthesis.

Several compounds have been prepared in this series. Keglevich and coworkers realized the synthesis of phosphole derivatives **2a–f** based on the McCormack reaction [10] followed by microwave-assisted esterification of the phosphinic acid using different alcohols in large excess (Scheme 1) [11–12]. Six phospholes **2a–f** were prepared in yields up to 94%.

**Scheme 2:**
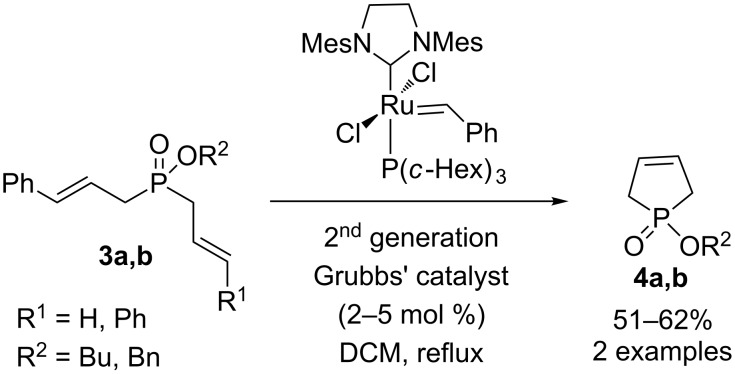
Ring-closing metathesis.

Montchamp and coworkers have synthesized phospholes **4a,b** by ring closing metathesis using 2 or 5 mol % of 2^nd^ generation Grubbs’ catalyst (Scheme 2) [13–14]. Two compounds **4a,b** were prepared in 51% and 62% yields. The same approach was reported earlier by Mioskowski and coworkers [15–16] except the starting phosphinates **3a,b** were prepared less efficiently by the sila-Arbuzov reaction of bis(trimethylsiloxy)phosphine (Me_3_SiO)_2_PH.

### Phosphindoles

**Scheme 3:**
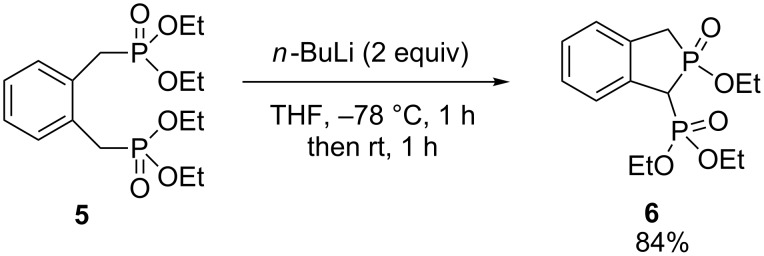
Phospha-Dieckmann condensation.

Montchamp and coworkers have synthesized a few phosphindoles. The first phosphindole **6** was simply obtained in 84% yield by reacting an α,ω-bisphosphonate derivative **5** with *n*-butyllithium in a phospha-Dieckmann condensation (Scheme 3) [17].

**Scheme 4:**
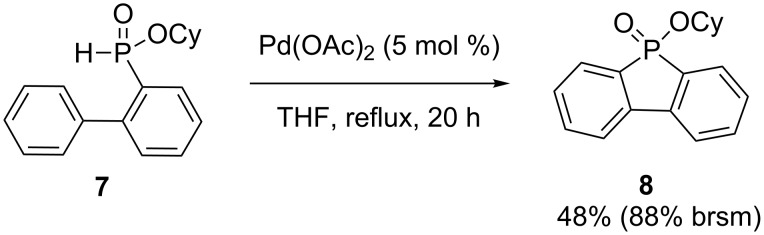
Palladium-catalyzed oxidative arylation.

Cyclohexyl 2-(biphenyl)-*H*-phosphinate **7** was cyclized using 2 mol % of Pd(OAc)_2_ in refluxing THF to produce another phosphindole **8** in 48% yield (Scheme 4) [18].

**Scheme 5:**
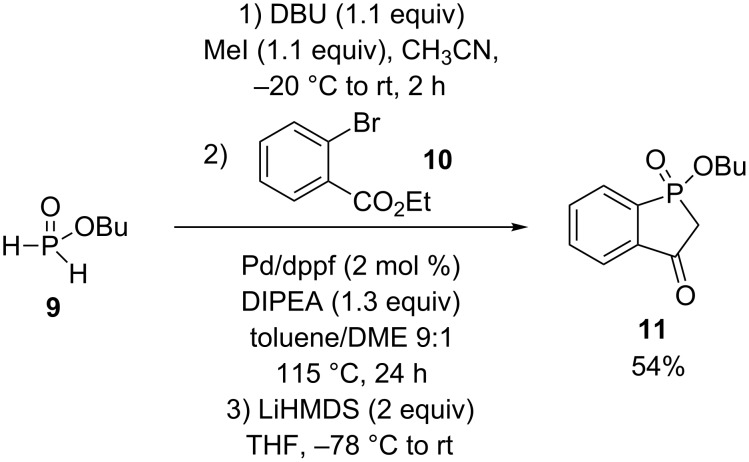
Tandem cross-coupling/Dieckmann condensation.

A phosphindol-3-one **11** was prepared in 54% yield from butylphosphinate **9** by first methylation using DBU and iodomethane followed by a cross-coupling with ethyl 2-bromobenzoate (**10**) and then a Dieckmann-like condensation using LiHMDS (Scheme 5) [19].

**Scheme 6:**
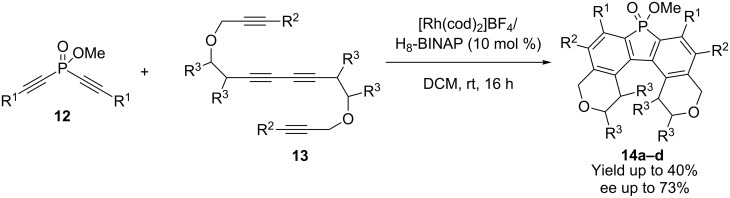
Rhodium-catalyzed double [2 + 2 + 2] cycloaddition.

Tanaka and coworkers have synthesized chiral benzopyrano and naphthopyrano-fused helical phosphafluorenes **14a–d** from dialkynyl phosphinate **12** and phenol-linked terminal tetrayne **13** at room temperature for only 1 h using a cationic rhodium(I)/(*R*)-tol-BINAP complex as a catalyst. Four helical phosphafluorenes **14a–d** were prepared in yields up to 40% and enantiomeric excesses up to 73% (Scheme 6) [20].

**Scheme 7:**
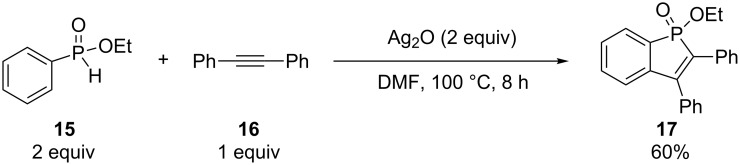
Silver oxide-mediated alkyne–arene annulation.

**Scheme 8:**
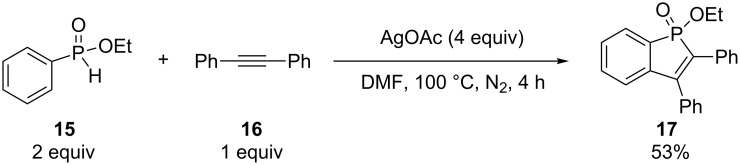
Silver acetate-mediated alkyne–arene annulation.

Chen and Duan have synthesized one phosphinoline **17** in 60% yield by the alkyne–arene annulation of ethyl phenyl-*H*-phosphinate (**15**) using 2 equivalents of Ag_2_O (Scheme 7) [21]. Miura et al. simultaneously reported the same reaction but with 4 equivalents of AgOAc instead, delivering the heterocycle **17** in 53% yield (Scheme 8) [22]. Both reactions used 4 equivalents of Ag(I) as well as an excess of *H*-phosphinate.

### 1,3-Oxaphospholes

**Scheme 9:**
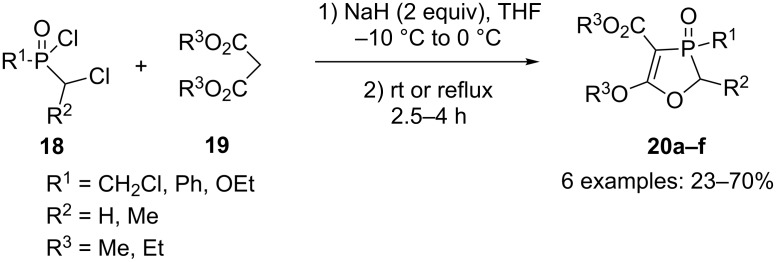
Cyclization through phosphinylation/alkylation of malonate anion.

Cristau and coworkers have achieved the direct synthesis of 1,3-oxaphospholes **20a–f** (Scheme 9) by reacting chloroalkylphosphinic or phosphonic chlorides **18** with malonic diester **19** in the presence of two equivalents of sodium hydride [23–24]. 1,3-Oxaphospholes **20a–f** were obtained in yields up to 70%.

### 1-Aza-3-phospha-6-oxabicyclo[3.3.0]octanes

**Scheme 10:**
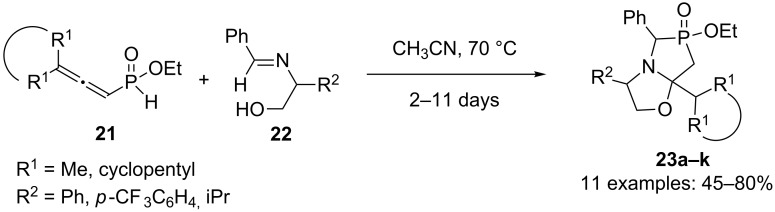
Tandem hydrophosphinylation/Michael/Michael reaction of allenyl-*H*-phosphinates.

The synthesis of chiral bicyclic phosphinates **23a–k** by domino hydrophosphinylation/Michael/Michael reaction was realized by Fourgeaud et al. (Scheme 10) [25].

Several 1-oxa-3-aza-6-phosphabicyclo[3.3.0]octanes derivatives **23a–k** were obtained in yields around 70% by reacting allenes **21** with imines **22** derived from (*R*)- or (*S*)-phenylglycinol, (*S*)-2-aminobutanol or ethanolamine. Diastereoisomeric ratios were generally close to 50:50. A model for this reaction’s diastereoselectivity was proposed.

### Cyclo-PALA

Montchamp and coworkers have achieved the synthesis of 5- and 6-membered rings “cyclo-PALA” analogs which are 1,3-azaphospholidine and 1,4-azaphosphorine derivatives **26**, **29** [26].

**Scheme 11:**
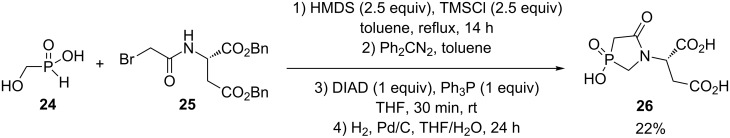
5-Membered “cyclo-PALA” via intramolecular Mitsunobu reaction.

For the 5-membered ring **26**, hydroxymethyl-*H*-phosphinic acid (**24**) underwent a sila-Arbuzov reaction with the bromide **25**, the crude mixture was esterified with diphenyldiazomethane, cyclized using Mitsunobu conditions and then hydrogenolyzed to produce the five-membered amide **26** in 22% overall yield (Scheme 11).

**Scheme 12:**
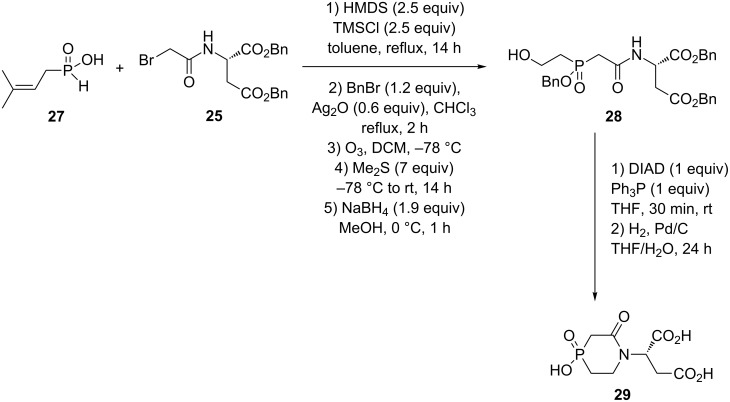
6-Membered “cyclo-PALA” via intramolecular Mitsunobu reaction.

For the six-membered “cyclo-PALA” **29**, isoprenyl-*H*-phosphinic acid (**27**) reacted with the bromide **25** under sila-Arbuzov conditions, the crude phosphinic acid was esterified, using BnBr/Ag_2_O, ozonolyzed and then reduced with sodium borohydride to afford an alcohol intermediate **28**. This product was cyclized using Mitsunobu conditions and finally hydrogenolyzed to deliver the 6-membered heterocycle **29** in 12% overall yield (Scheme 12) [26].

In this particular study phosphinates **26** and **29** were tested as inhibitors of aspartate transcarbamoylase (ATCase). 5-Membered **26** was completely inactive, whereas 6-membered **29** showed modest activity (*K*_i_ = 1 μM, 63 times less active than phosphonic acid *N*-phosphonacetyl-L-aspartate PALA, *K*_i_ = 16 nM).

### 1,3-Azaphosphorines and 1,3-azaphospholidines

**Scheme 13:**
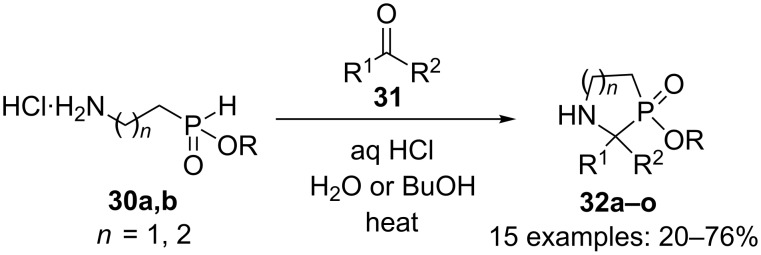
Intramolecular Kabachnik–Fields reaction.

Several 1,3-azaphosphorines and 1,3-azaphospholidines were synthesized by Montchamp and coworkers*.* The reaction of 2-aminoethyl-*H*-phosphinate **30a** (*n* = 1) with carbonyl compounds **31** in refluxing butanol or concentrated hydrochloric acid took place smoothly to generate seven 1,3-azaphospholidines **32a–g** in yields up to 55% (Scheme 13) [27–28].

The reaction of 3-aminopropyl-*H*-phosphinate **30b** with aldehydes **31** in refluxing butanol allowed the formation of eight 1,3-azaphosphorines **32h–o** in yields up to 76% (Scheme 13).

**Scheme 14:**
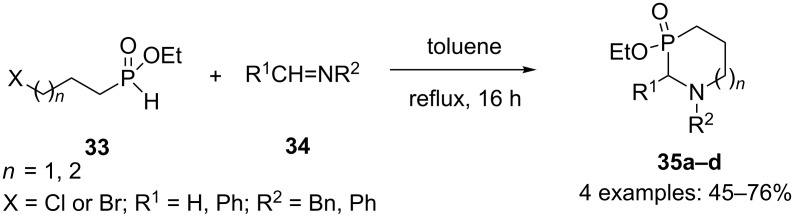
Tandem Kabachnik–Fields/alkylation reaction.

Montchamp and coworkers also prepared two other examples of 1,3-azaphosphorines **35a,b** (*n* = 1) in yields up to 61% by reacting ethyl-3-chloropropyl-*H*-phosphinate **33** with imines **34** in toluene at reflux (Scheme 14) [29].

### 1,3-Azaphosphindoles and 1,3-benzazaphosphorines

**Scheme 15:**
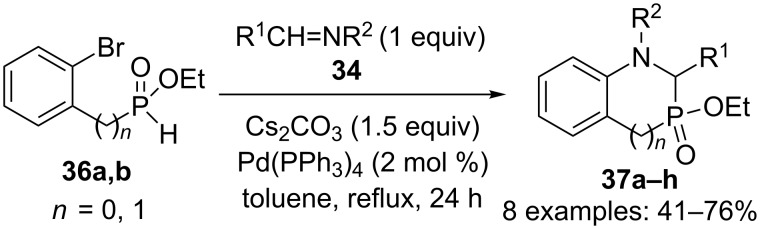
Tandem Kabacknik–Fields/C–N cross-coupling reaction.

Several compounds in this series were synthesized by Montchamp and coworkers using two different approaches. The first one is the reaction between an imine **34** and 2-bromophenyl-substituted *H*-phosphinate esters **36** in the presence of Cs_2_CO_3_, and catalytic Pd(PPh_3_)_4_ in refluxing toluene to generate the corresponding cyclized products **37a–h** in yields up to 76% (Scheme 15) [29].

**Scheme 16:**
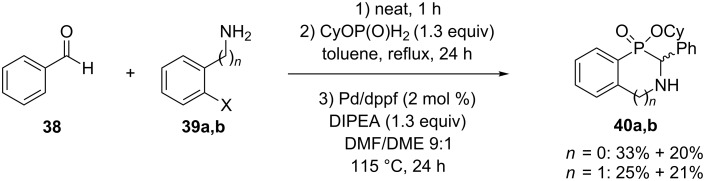
Tandem Kabacknik–Fields/C-P cross-coupling reaction.

The second way is the formation of the imine first by reacting an amine **39a,b** with an aldehyde **38**, then the phosphinate is introduced and the mixture stirred for 24 h at reflux to generate the corresponding *H*-phosphinate esters. Addition of DIPEA and catalytic Pd/dppf in a mixture DMF/DME to the intermediates generated the corresponding cyclized derivatives **40a,b** in yields up to 53% (Scheme 16) [18].

For these compounds, the authors were able to separate the different diastereoisomers generated during the reaction by simple column chromatography on silica gel.

### 1,4-Azaphosphorines

**Scheme 17:**
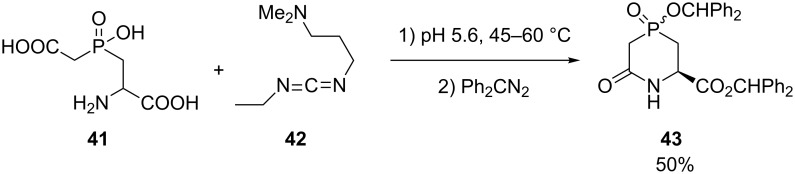
Heterocyclization via amide formation.

In this series, only a few examples have been reported in the literature. One derivative has been prepared by Manthey and coworkers in 50% yield as a precursor to a dihydroorotase inhibitor (Scheme 17) [30]*.*

In this example, the amino acid **41** was first cyclized using 1-(3-dimethylaminopropyl)-3-ethylcarbodiimide (**42**) at pH 5.6 followed by protection of the carboxylic acid and phosphinic acid moieties by diphenylmethyl group using a slight excess of diphenyldiazomethane. The two diastereoisomers obtained were readily separable by column chromatography.

**Scheme 18:**
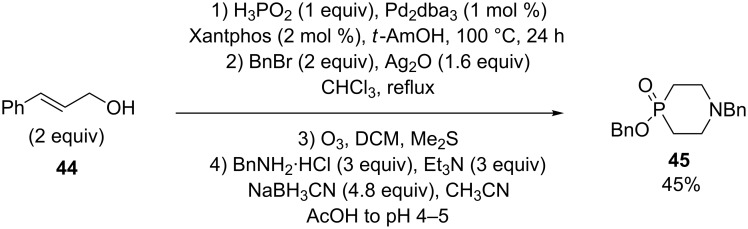
Cyclization via reductive amination.

Another example has been synthesized in 45% yield by Montchamp and coworkers (Scheme 18) [14]*.*

To prepare the required phosphinate **45** a double allylation of H_3_PO_2_ was performed using 2 equivalents of cinnamyl alcohol **44** in the presence of 2 mol % of Pd/Xanthpos followed by an esterification using benzyl bromide. Ozonolysis, and reductive amination using excess benzylamine in the presence of sodium cyanoborohydride completed the synthesis.

### Phosphorines

**Scheme 19:**
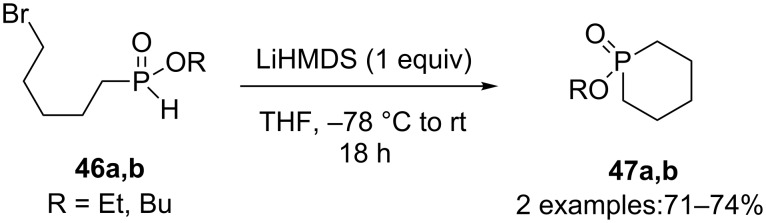
*H*-Phosphinate alkylation.

Two phosphorines **47a,b** were obtained by Montchamp and coworkers via the cyclization of 5-bromopentyl-*H*-phosphinate esters **46a,b** in the presence of LiHMDS in 71% and 74% yields for the butyl and ethyl esters respectively (Scheme 19) [28,31].

**Scheme 20:**
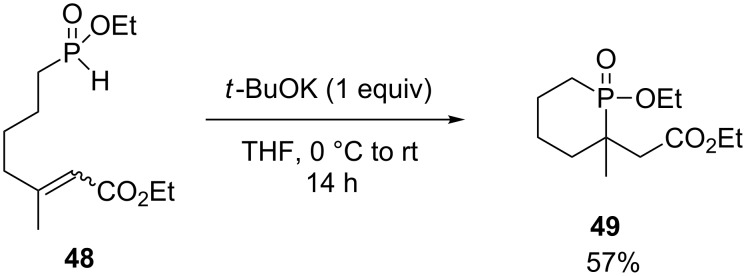
Cyclization through intramolecular Michael addition.

Another phosphorine **49** was obtained by Montchamp and coworkers in 57% yield via the cyclization through conjugate addition of ethyl 7-(ethoxy-*H*-phosphinoyl)-3-methyl-2-heptenoate (**48**) in the presence of potassium *tert*-butoxide (Scheme 20) [28].

**Scheme 21:**
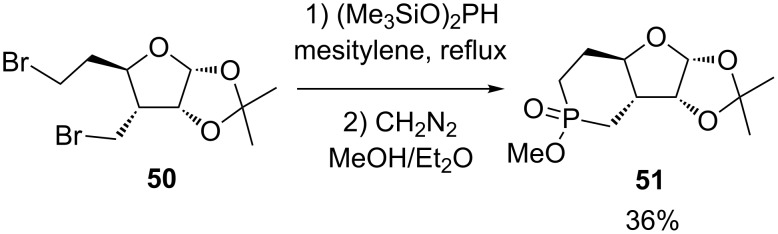
Double Arbuzov reaction of bis(trimethylsiloxy)phosphine.

A phosphorino[3’,4’:4,5]furo[2,3-*d*]-1,3-dioxole **51** was synthesized in 36% yield by Tattersall and coworkers by realizing a double Arbuzov-type reaction between bis(trimethylsiloxy)phosphine and the dibromide **50** followed by the esterification of the phosphinic acid using diazomethane (Scheme 21) [32]. The heterocyclization step followed methodology initially introduced by Frost et al [33].

Compound **51** was subsequently converted into the corresponding analog of cyclic AMP, but no biological activity was reported.

### 1,2-Oxaphosphorines

**Scheme 22:**
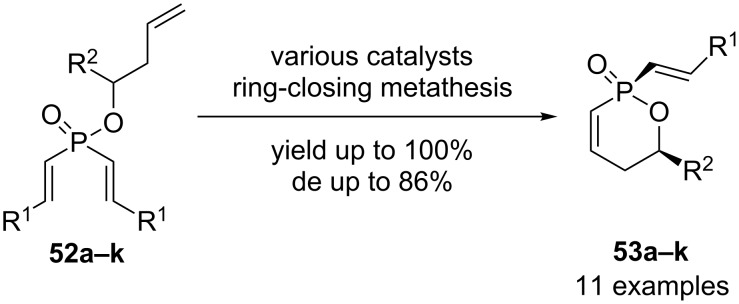
Diastereoselective ring-closing metathesis.

Gouverneur and coworkers have realized the synthesis of several 1,2-oxaphosphorine derivatives **53a–k** using diastereoselective ring closing metathesis with 2 to 4 mol % of various catalysts (Scheme 22) [34].

During this work, they obtained 11 different compounds in yields up to 100% and diastereomeric excesses up to 86%. The starting phosphinates **52a–k** were prepared using classical chemistry involving Grignard addition to EtOP(O)Cl_2_.

### Phenoxaphosphine

**Scheme 23:**
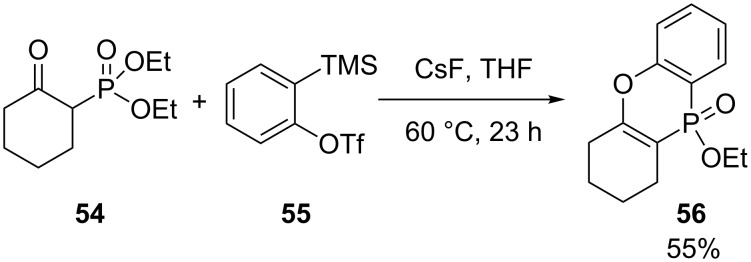
2-Ketophosphonate/benzene annulation.

Scheme 23 shows the synthesis of one phenoxaphosphine **56** in 55% yield by Li and coworkers via the reaction between diethyl 2-oxocyclohexylphosphonate (**54**) and benzyne generated from 2-(trimethylsilyl)phenyl triflate (**55**) and cesium fluoride [35].

### 1,4,2-Oxazaphosphinane

**Scheme 24:**
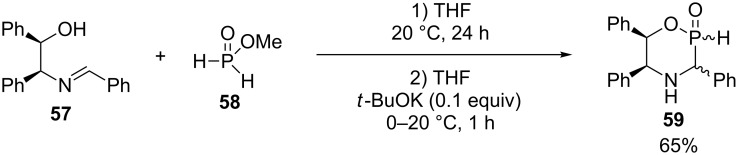
Tandem Kabachnik–Fields/transesterification reaction.

This series of compounds is only represented by few examples all generated through methodology developed by Pirat and coworkers. Scheme 24 shows the synthesis of a *H*-phosphinate intermediate **59** in 65% yield via the reaction between the imine **57** of the racemic 1,2-diphenylethanolamine with benzaldehyde and methyl phosphinate (**58**) followed by the cyclization through a base catalyzed transesterification [23,36].

This versatile intermediate **59** was reacted with aldehydes, imines, olefins and aryl bromides or aryl iodides to generate a wide range of phosphinates.

**Scheme 25:**
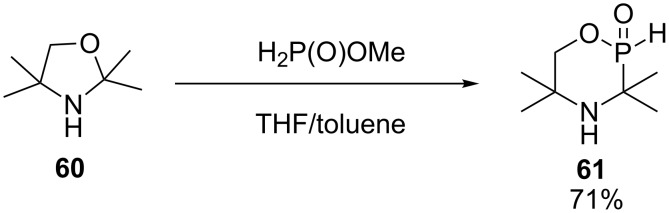
Tandem Kabachnik–Fields/transesterification reaction with oxazolidine.

The same authors have also prepared another *H*-phosphinate intermediate **61** in 71% yield (Scheme 25) [37].

This oxazaphosphinane **61** was synthesized in two steps at room temperature, first, by a nucleophilic attack of methyl hypophosphite on oxazolidine **60** followed by an intramolecular cyclization, this time without base catalyzed transesterification. The authors explained this difference of reactivity by the Thorpe–Ingold effect [38]. Indeed, the presence of four methyl groups allows the hydroxy function to be spatially closer to the reactive phosphinate, facilitating the intramolecular cyclization of this product.

## Conclusion

Phosphinate heterocycles are becoming routine products in the literature. Classical approaches such as the McCormack reaction of conjugated dienes, the sila-Arbuzov reaction of bis(trimethylsiloxy)phosphine with dihalides, etc. continue to be useful. However, novel approaches in both the preparation of acyclic precursors and the reactions to achieve their heterocyclization, have led to more efficient synthesis and broader structural diversity. While, like with any other P-heterocycles the phosphinates can be employed for the synthesis of novel phosphine ligands, their potential for the discovery of novel biologically active motifs is tantalizing.
